# Supplementary material for: Distinct microbial communities are linked to organic matter properties in millimetre-sized soil aggregates
Source: ISME J. 2024 Aug 6;18(1):wrae156. doi: 10.1093/ismejo/wrae156 (PMC11325450; doi:10.1093/ismejo/wrae156)
Supplement: Textsummary_Supplementary_Material_wrae156 [file textsummary_supplementary_material_wrae156.docx]

Text Summary, Supplementary Material

The Supplementary Material file (.docx) contains 6 supplementary figures and 3 supplementary tables. They either provide relevant additional information for the Material and Methods section or support claims in the Discussion section of our paper.

Furthermore, we include a Supplementary Material and Methods section describing soil sampling in the field, core transport to the lab and aggregate sampling and handling in detail.
